# A head-to-head comparison of fast-SENC and feature tracking to LV long axis strain for assessment of myocardial deformation in chest pain patients

**DOI:** 10.1186/s12880-022-00886-3

**Published:** 2022-09-05

**Authors:** Deborah Siry, Johannes Riffel, Janek Salatzki, Florian André, Lukas Damian Weberling, Marco Ochs, Noura A. Atia, Elizabeth Hillier, David Albert, Hugo A. Katus, Evangelos Giannitsis, Norbert Frey, Matthias G. Friedrich

**Affiliations:** 1 Department of Cardiology, Angiology and Pneumology, University Clinic of Heidelberg, Heidelberg, Germany; 2 Department of Cardiology and Angiology, Robert-Bosch-Hospital, Stuttgart, Germany; 3 Department of Cardiology, Angiology and Internal Intensive Care, Theresien-Hospital, Mannheim, Germany; 4 Diagnostic Radiology and Medical Imaging Department, Faculty of Medicine, Tanta University, Tanta, Egypt; 5 Departments of Medicine and Diagnostic Radiology, McGill University Health Centre, Montreal, QC Canada; 6 DZHK (German Centre for Cardiovascular Research), Partner Site Heidelberg, Heidelberg, Germany

**Keywords:** Myocardial strain, Fast-SENC, Feature tracking, LV long axis strain

## Abstract

**Background:**

Myocardial strain imaging has gained importance in cardiac magnetic resonance (CMR) imaging in recent years as an even more sensitive marker of early left ventricular dysfunction than left-ventricular ejection fraction (LVEF). fSENC (fast strain encoded imaging) and FT (feature tracking) both allow for reproducible assessment of myocardial strain. However, left-ventricular long axis strain (LVLAS) might enable an equally sensitive measurement of myocardial deformation as global longitudinal or circumferential strain in a more rapid and simple fashion.

**Methods:**

In this study we compared the diagnostic performance of fSENC, FT and LVLAS for identification of cardiac pathology (ACS, cardiac-non-ACS) in patients presenting with chest pain (initial hscTnT 5–52 ng/l). Patients were prospectively recruited from the chest pain unit in Heidelberg. The CMR scan was performed within 1 h after patient presentation. Analysis of LVLAS was compared to the GLS and GCS as measured by fSENC and FT.

**Results:**

In total 40 patients were recruited (ACS n = 6, cardiac-non-ACS n = 6, non-cardiac n = 28). LVLAS was comparable to fSENC for differentiation between healthy myocardium and myocardial dysfunction (GLS-fSENC AUC: 0.882; GCS-fSENC AUC: 0.899; LVLAS AUC: 0.771; GLS-FT AUC: 0.740; GCS-FT: 0.688), while FT-derived strain did not allow for differentiation between ACS and non-cardiac patients. There was significant variability between the three techniques. Intra- and inter-observer variability (OV) was excellent for fSENC and FT, while for LVLAS the agreement was lower and levels of variability higher (intra-OV: Pearson > 0.7, ICC > 0.8; inter-OV: Pearson > 0.65, ICC > 0.8; CoV > 25%).

**Conclusions:**

While reproducibility was excellent for both FT and fSENC, it was only fSENC and the LVLAS which allowed for significant identification of myocardial dysfunction, even before LVEF, and therefore might be used as rapid supporting parameters for assessment of left-ventricular function.

## Background

Strain imaging has gained importance in cardiac magnetic resonance (CMR) imaging in recent years. It allows for very sensitive assessment of myocardial deformation and has been shown to detect subclinical changes in patients with a variety of underlying cardiac conditions such as hypertrophic cardiomyopathy, cardiotoxic cancer therapy patients, ischemic heart disease, heart failure, atrial fibrillation, hypertension, or valvular heart disease [1]. Furthermore, recent data has shown promising results regarding myocardial strain for prognostic information [2].

Several methods exist for strain measurement: recent more rapid techniques include fast-strain encoded imaging (fSENC), feature tracking (FT) and left-ventricular long axis strain (LVLAS).

fSENC uses tag lines that are oriented parallel to the imaging plane from which color-coded images can be generated [3].

A disadvantage of fSENC imaging is the non-measurable radial strain and the need for a separate image acquisition sequence and assessment software. However, it has been proven to be highly reproducible [4] and allows for differentiation between different degrees of infarcted tissue [5]. The advantages of fSENC are its objectivity, reproducibility, short breath-hold times and fast post-processing times[6, 7]. Furthermore, we could recently demonstrate the feasibility of fSENC within a patient population presenting with new onset of chest pain [8].

FT enables the measurement of strain by tracing anatomic elements along the cavity-myocardial interface in cine images. Therefore, no additional images need to be acquired and retrospective analyses of pre-existing datasets can be performed. A disadvantage, however, is its susceptibility to through-plane motion artefacts and partial volume effects [9]. However, it has been shown to detect infarcted territories quite accurately, even allowing discrimination between subendocardial or transmural infarction [10] while remaining highly reproducible [11].

A recently emerging parameter for LV functional assessment is the LVLAS. It has been shown that the major portion of stroke volume is generated by the longitudinal atrioventricular plane movement [12]. As suggested by Riffel et al. the LVLAS is measured as the fractional change in distance between epicardial LV apex and the midpoint between the origin of both mitral valve leaflets calculated in end systole and end diastole [13]. Recent studies suggest that LVLAS is an independent and reproducible predictor for adverse cardiac events in patients with cardiac pathology [14–17].

All these different strain imaging techniques have their individual advantages and pitfalls, yet no direct comparison of LVLAS to FT/fSENC has been performed so far.

The purpose of this study was to assess and directly compare both GLS (global longitudinal strain) and GCS (global circumferential strain) using fSENC and FT as well as LVLAS within a study population of patients presenting with chest pain. We aimed to explore the diagnostic performance of the different strain techniques for identification of myocardial dysfunction (acute coronary syndrome (ACS) or underlying cardiac pathologies) as well as evaluate correlation of GLS and GCS between the three strain imaging tools.

Furthermore, we hypothesized that LVLAS is comparable to fSENC or FT for LV-function assessment and offers a rapid analysis of myocardial deformation in a simpler and clinically applicable fashion.

## Methods

### Study population

Patients were prospectively recruited from the chest pain unit of the University Hospital in Heidelberg using a randomized double-blinded single-center study design (consecutive sampling). The inclusion and exclusion criteria for patient enrolment is provided in Table 1.

**Table 1:** Inclusion and exclusion criteria

Inclusion criteria	Exclusion criteria
Chest pain HEART score ≤ 6 hscTnT 5–52 ng/l (0 h/1 h algorithm) Signed informed consent	Acute ST-elevation myocardial infarction Hemodynamic instability Systolic heart failure (LVEF < 40%) Atrial fibrillation/frequent extrasystoles Stent implants/bypass operation Non-suitable metallic implants for CMR Severe claustrophobia

The CMR scan was performed within a one-hour timeframe before the 2nd hscTnT measurement [18]. Patients were closely monitored (ECG, pulse oximetry, accompanied by a physician during in-hospital transport) at all times. The study was approved by the local ethics committee (Ethikkommission Medizinische Fakultät Heidelberg (S-483/2018)). All participants provided informed written consent and all methods were performed in accordance with the relevant guidelines and regulations.

### CMR acquisition

CMR scans were all performed in a 1.5 Tesla whole-body CMR scanner (Ingenia CX 1.5 T, Philips Medical Systems, Best, The Netherlands). A vector ECG was applied for R-wave triggering.

The study protocol included:

- Standard SSFP cine function images: long axis (LAX) (2, 3 and 4 chamber) and short axis (SAX) (apical, mid, basal)
  (FOV 140 mm^2^, TE 1.38 ms, TR 2.77 ms, flip angle 60°, pixel size 0.88 × 0.88 mm^2^, 35 acquired phases, slice thickness 8 mm)
- fSENC images planned on an end systolic timeframe: LAX (2, 3 and 4 chamber) and SAX (apical, mid, basal)
  (FOV 100 mm^2^, TE 0.71 ms, TR 12.16 ms, flip angle 30°, pixel size 1 × 1 mm^2^, slice thickness 10 mm)

### CMR analysis

**Fig. 1:**
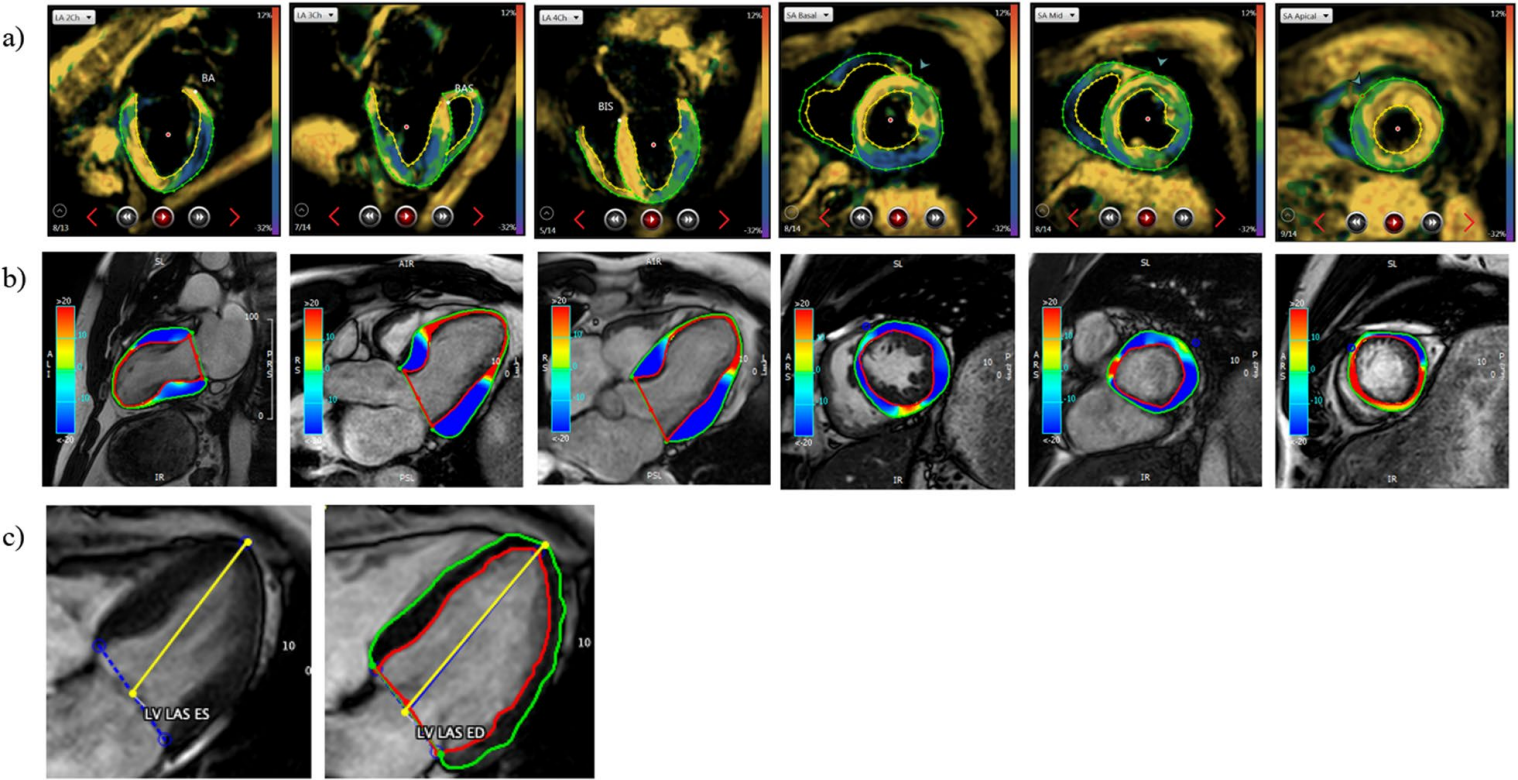
**a** fSENC manual contouring in end systole (endocardial and epicardial borders) in 2-CH, 3-CH, 4-CH long-axis views and basal, midventricular, apical short-axis views. **b** FT contouring in 2-CH, 3-CH, 4-CH long-axis views and basal, midventricular, apical short-axis views. **c** LVLAS as fractional change in length between the epicardial tip to the middle of a line connecting mitral valve leaflet origins between end systole and end diastole ((LVLAS-ES-LVLAS-ED)/LVLAS-ED*100)

For FT the software “cvi42” (Circle Cardiovascular Imaging Inc., Calgary, AB, Canada) was applied. Using the “Tissue Tracking” module, GCS and GLS were semi-automatically calculated by averaging peak strain values of individual segments based on the 16-segment model. We also performed a sub-analysis based on artificial intelligence (AI) automated contouring (Fig. 1b).

fSENC images were semi-automatically analyzed after manual contouring of endocardial and epicardial borders (papillary muscles, trabeculae and epicardial fat excluded from blood pool) by the software Myostrain® (Myocardial Solutions, Morrisville, NC, USA). GCS and GLS were reported and represented in color-coded bull’s-eye plots according to the American Heart Association 16-segment model. Longitudinal strain was derived from the SAX images, whereas circumferential strain was gained from the LAX images (Fig. 1a).

LVLAS was calculated in a standard 4-chamber view by measuring the difference in length between the apex to the midpoint of the origin of both mitral valve leaflets between end systole (ES) and end diastole (ED) using the following formula (Fig. 1c):

$${\text{LVLAS}} = ({\text{LVLASES}}-{\text{LVLASED}})/{\text{LVLASED}}*100$$

### Intra- and interobserver reproducibility

20 scans for FT and 15 scans for fSENC as well as LVLAS measurement were randomly selected and separately analyzed a second time by the lead study investigator after a period of more than 6 months (no recall bias) as well as another unbiased investigator. All observers are certified experienced readers and were blinded to patients’ final clinical diagnoses.

### Reference standard

The reference standard was based on the patient’s final clinical diagnosis as determined by staff cardiologists blinded to our results. Final diagnosis was derived from serial hscTnT testing (4th generation cTnT assay, Roche Diagnostics, Penzberg, Germany [19]) and, if clinically indicated, further diagnostic procedures such as coronary angiography, echocardiography, coronary CT, standard stress CMR or stress ECG.

### Statistics

The primary endpoint of this study was to assess the diagnostic performance of LVLAS in comparison to the more established parameter GLS.

Therefore, the null hypothesis was formulated as H_0_: LVLAS is less accurate at identifying cardiac dysfunction in patients with chest pain than GLS by fSENC and FT.

For all statistical analyses the software programs Excel (Microsoft, Redmond, CA, USA), SPSS (Version 24, IBM, Armonk, USA) and MedCalc (Version 19.2, MedCalc Software, Ostend, Belgium) were used.

Quantitative data is represented with mean values and standard deviation (SD). Receiver Operating Characteristic (ROC) curves were calculated and the area under the curve (AUC) was determined. ROC curves were compared using the Hanley and McNeil test [20]. The data was analyzed using student’s t-test for independent samples and displayed in boxplots. Correlation analysis, intra- and inter-observer analyses were assessed using Pearson’s correlation coefficient as well as intraclass correlation coefficient (ICC). Linear regression analyses and Bland–Altman plots with 95% confidence intervals were drawn to determine levels of bias. P-values < 0.05 were regarded as statistically significant.

## Results

### Reference standard, study duration

In total, we prospectively recruited 40 patients with chest pain. Of these 40 patients 6 were found to have ACS (n = 3 non-ST-elevation myocardial infarction), another 6 had an underlying cardiac, non-ACS disease (n = 4 septal hypertrophy/hypertensive heart disease; n = 2 hypertrophic cardiomyopathy), while the remaining 28 were determined to have no cardiac cause of the chest pain. Patient characteristics are depicted in Table 2a, b. Gender was evenly distributed (n = 20 female; n = 20 male) with a mean age of 57.1 ± 17.7 years.

**Table 2:** (a) Patient characteristics and (b) Patient characteristics according to underlying diagnosis

Total: 40		Count	Mean ($\pm$ SD)	max/min
(a)				
Sex	Female	20		
	Male	20		
Age (years)			57.1 ± 17.7	84/23
BMI (kg/m^2^)			26.4 $\pm$ 3.7	34.4/18.9
BP (systolic) (mmHg)			158 $\pm$ 23	204/117
HR (bpm)			74 $\pm$ 14	104/43
HEART score	Low		14	
	Intermediate		26	
NYHA	1	32 (80%)		
	2	3 (7.5%)		
	3	5 (12.5%)		
	4	0 (0%)		
EF (%)			72.4 $\pm$ 11.6	
EDV (ml)			114.6 $\pm$ 44.1	
ESV (ml)			32.5 $\pm$ 22.7	
Diabetes		2 (5%)		
Hypertension		18 (45%)		
Hypercholesterinemia		9 (22.5%)		
Familial predisposition		12 (30%)		
nicotine (py)	Non-smoker	22 (55%)	0 $\pm$ 0	0/0
	Past smoker	13 (32.5%)	19.5 $\pm$ 15.4	45/2
	Smoker	5 (12.5%)	17.8 $\pm$ 12.8	45/4
hscTnT 0 h (ng/L)			10.8 $\pm$ 7.0	32/5
hscTnT 1 h (ng/L)			15.9 $\pm$ 19.9	88/3
Diagnostic procedures	stress ECG	2 (5%)		
	echocardiography	2 (5%)		
	standard CMR	1 (2.5%)		
	CT angiography	1 (2.5%)		
	coronary angiography	11 (27.5%)		

	Group 0: non-cardiac	Group 1: ACS	Group 2: cardiac, non-ACS	
(b)				
Sex (count)	f: n = 17; m: n = 11	f: n = 1; m: n = 5	f: n = 2; m: n = 4	
Age (mean $$\pm$$ SD $$)$$	54.8 $$\pm$$ 18 years	68.3 $$\pm$$ 13 years	56.8 $$\pm$$ 18 years	
hscTnT 0 h (mean $\pm$ SD)	8.5 $\pm$ 5 ng/L	16.5 $\pm$ 9 ng/L	14.2 $\pm$ 9 ng/L	
hscTnT 1 h (mean $\pm$ SD)	8.7 $\pm$ 7 ng/L	40.0 $\pm$ 36 ng/L	15.3 $\pm$ 7 ng/L	
EF (mean $\pm$ SD)	72.3 $\pm$ 10%	74.9 $\pm$ 19%	69.6 $\pm$ 9%	
LVESV (mean $\pm$ SD)	29.5 $\pm$ 17 ml	35.1 $\pm$ 39 ml	44.1 $\pm$ 25 ml	
LVEDV (mean $\pm$ SD)	104.4 $\pm$ 36 ml	129.4 $\pm$ 51 ml	145.8 $\pm$ 57 ml	

max: maximum, min: minimum, SD: standard deviation, BMI: body mass index, BP: blood pressure, HR: heart rate, NYHA: New York Heart Association, EF: ejection fraction, ESV: End-systolic volume, EDV: End-diastolic volume, py: pack years, h: hours, ACS: acute coronary syndrome, hscTNT: high-sensitive cardiac troponin T, ECG: electrocardiogram, CMR: cardiovascular magnetic resonance, CT: computed tomography, SD: standard deviation, EF: ejection fraction, ESV: End-systolic volume, EDV: End-diastolic volume,, h: hours, ACS: acute coronary syndrome, hscTNT: high-sensitive cardiac troponin T

All CMR scans were performed with a mean study time of 19.5 ± 5.3 min, including patient preparation and scan time.

### ROC curve analysis

ROC curves were drawn for differentiation between cardiac pathology (6 ACS patients and 6 patients with underlying cardiac disease) from non-cardiac chest pain (n = 28).

**Fig. 2:**
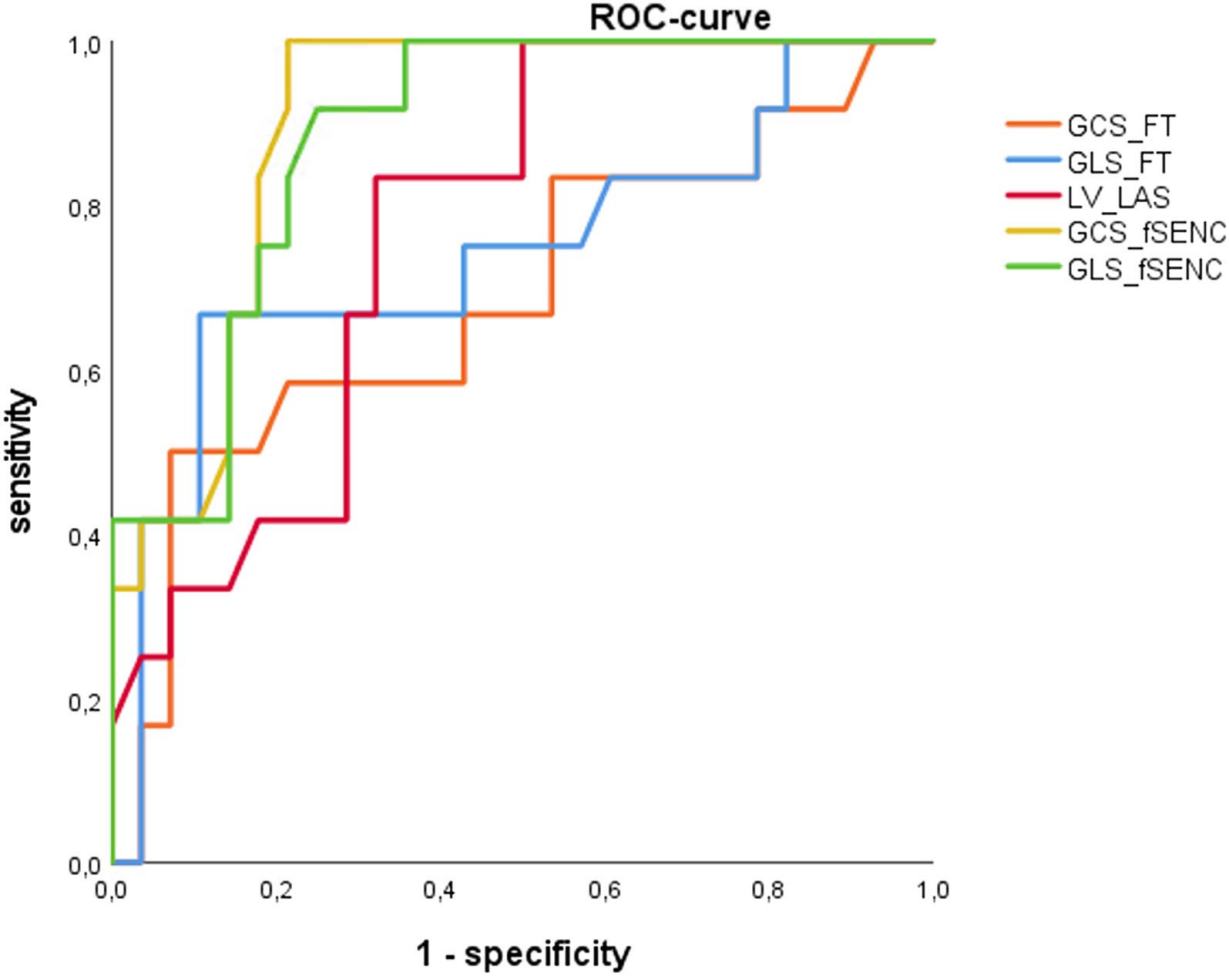
ROC curve: identification of cardiac pathology (ACS n = 6/cardiac, non-ACS n = 6) (GCS-fSENC AUC:0.899, GLS-fSENC AUC: 0.882, LVLAS AUC: 0.771, GCS-FT AUC: 0.688, GLS-FT AUC: 0.740)

GCS-fSENC proved to be the strongest parameter for identification of cardiac dysfunction within the study population (AUC: 0.899) closely followed by GLS-fSENC (AUC: 0.882). Notably, LVLAS achieved good results with an AUC of 0.771, while FT strain values demonstrated the weakest performance (GCS-FT AUC: 0.688, GLS-FT AUC: 0.740). GCS-FT differed significantly from GCS- and GLS-fSENC curves (GCS-FT vs. GCS-fSENC *p* < 0.025; GCS-FT vs. GLS-fSENC *p* < 0.035), whereas for the other parameters the difference was not significant (Fig. 2).

### Triage analysis

**Fig. 3:**
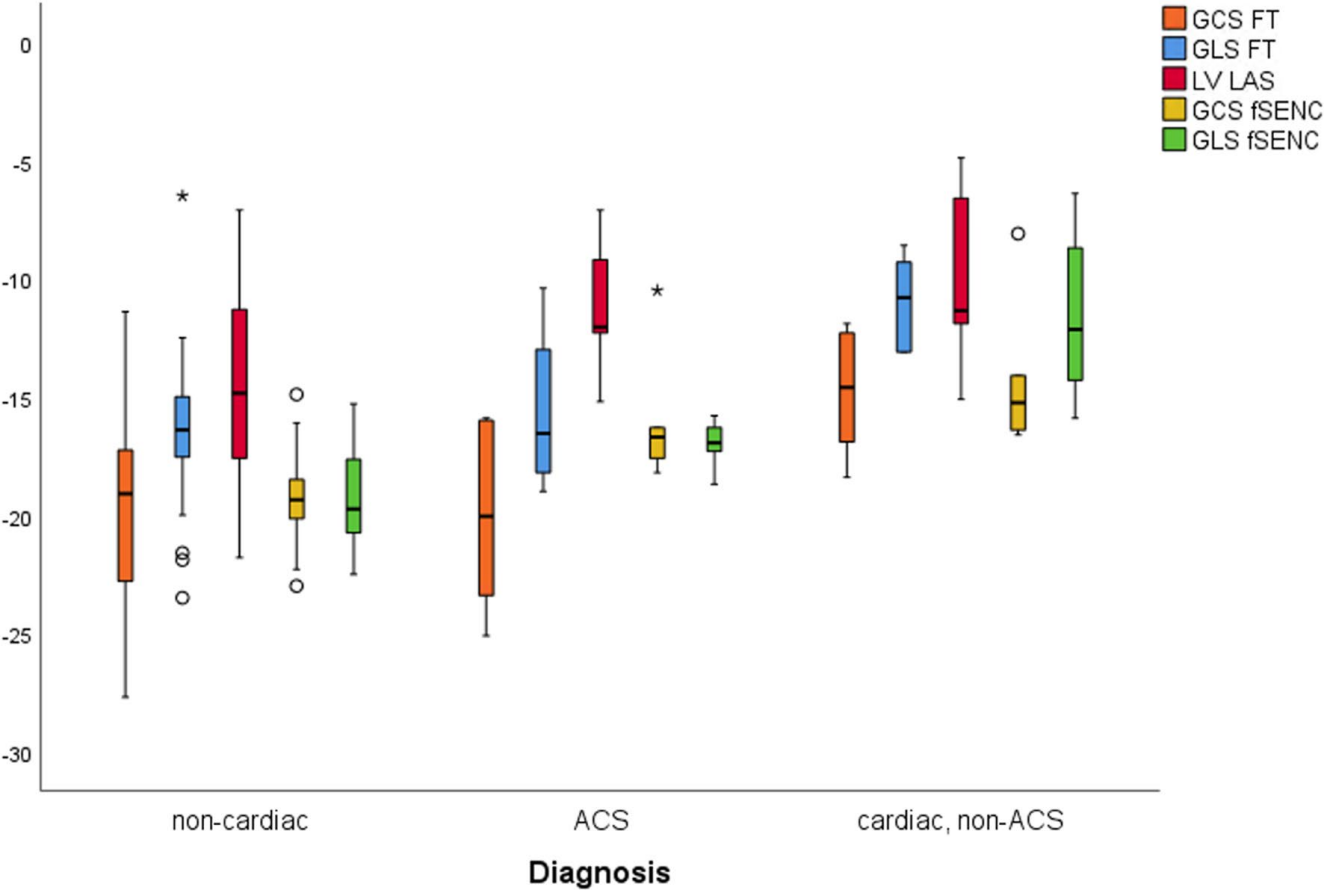
Boxplots of GCS-FT, GCS-fSENC, GLS-FT, GLS-fSENC, LVLAS. Significant difference (*p* < 0.05) for (1) differentiation between non-cardiac and cardiac, non-ACS for fSENC/FT/LVLAS (2) differentiation between non-cardiac and ACS for fSENC and LVLAS (3) differentiation between ACS and cardiac, non-ACS for FT and GLS-fSENC

In a further analysis patients were triaged according to diagnosis (0: non-cardiac/1: ACS/2: cardiac-non-ACS) and the GLS and GCS (fSENC, FT) as well as LVLAS compared between the three patient groups. Results are depicted in Fig. 3 and Tables 3 and 4.

**Table 3:** Mean ± standard deviation (SD) with 95% confidence interval (CI) and p-values for all deformation parameters within total study population

	FT	fSENC
GLS (%)	−15.47 ± 3.63 (95% CI −16.63 to −15.47; *p* < 0.001)	−17.82 ± 3.25 (95% CI −16.93 to −18.70; *p* < 0.001)
GCS (%)	−19.11 ± 3.99 (95% CI −20.39 to −17.84; *p* < 0.001)	−17.22 ± 5.53 (95% CI −15.71 to −18.73; *p* < 0.001)
LVLAS	−13.42 ± 3.87 (95% CI −12.18 to −14.65; *p* < 0.001)

**Table 4:** p-values for triage analysis (group 0: non-cardiac, group 1: ACS, group 2: cardiac, non-ACS) according to strain parameters

	Group 0 versus 1	Group 0 versus 2	Group 1 versus 2
GLS-fSENC	*p* < 0.05*	*p* < 0.001***	*p* < 0.005**
GCS-fSENC	*p* < 0.005**	*p* < 0.001***	*p* = 0.344
LVLAS	*p* < 0.05*	*p* < 0.05*	*p* = 0.573
GLS-FT	*p* = 0.59	*p* < 0.001***	*p* < 0.05*
GCS-FT	*p* = 0.88	*p* < 0.005**	*p* < 0.05*

*p < 0.05;**p < 0.005;***p < 0.001

All strain parameters could significantly (fSENC/FT: *p* < 0.005; LVLAS: *p* < 0.02) differentiate between non-cardiac (0) and underlying cardiac disease patients (2).

While GCS-fSENC (*p* < 0.0025), GLS-fSENC (*p* < 0.025) and LVLAS (*p* < 0.05) all allowed for significant differentiation between non-cardiac (0) and ACS patients (1), further separation between ACS (group 1) and other cardiac diseases (group 2) was only possible with GLS-fSENC (*p* < 0.006). GCS- and GLS-FT while not allowing for distinction between non-cardiac (0) and ACS (1) patients, were significantly different between ACS (1) and cardiac, non-ACS (2) patients (*p* < 0.02).

### Correlation

**Fig. 4:**
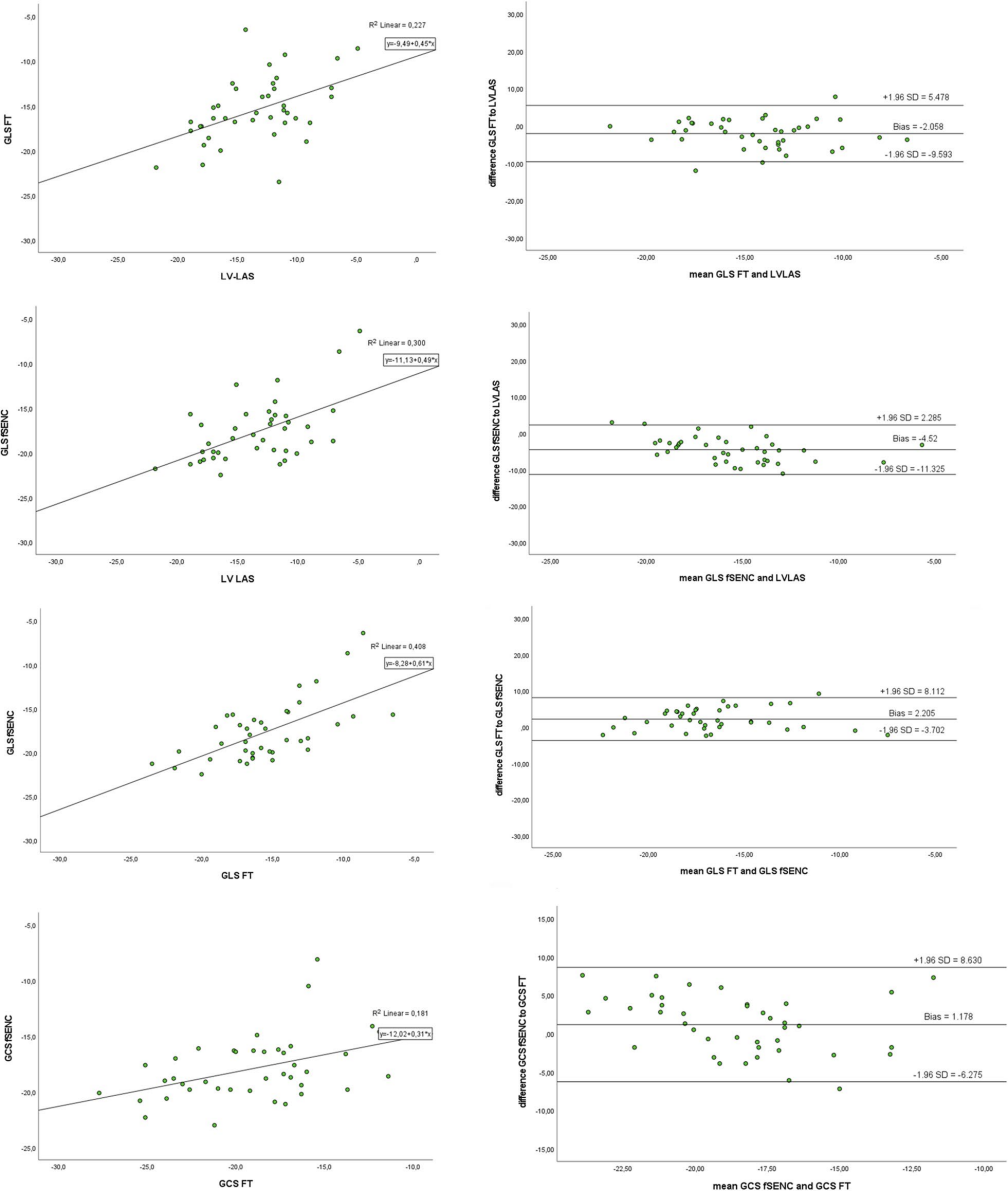
Linear regression analysis and Bland–Altman plots for GLS values (derived by FT/fSENC) compared to LVLAS and to each other as well as to GCS values (derived by FT/fSENC)

Correlation coefficients (Pearson and ICC) for the different myocardial deformation parameters are given in Tables 5, 6 and 7. Pearson’s correlation coefficient was notably strong (> 0.5) between GLS-FT and GLS-fSENC as well as between GCS-/GLS-fSENC and LVLAS. The ICC values were good (> 0.75) between GLS-FT and GLS-fSENC. All correlation values were statistically significant (*p* < 0.05). Linear regression analyses are depicted graphically in scatter plots (Fig. 4) showing a weak linear relationship between GLS as derived by fSENC or FT and compared to the LVLAS (R^2^ < 0.5). The correlation was strongest, albeit weak, between GLS-fSENC and GLS-FT (R^2^ = 0.408).

**Table 5:** Pearson’s correlation coefficient for all deformation parameters

	GCS-FT	GLS-FT	LVLAS	GCS-fSENC	GLS-fSENC
GCS-FT	1	0.754**	0.330*	0.426**	0.468**
GLS-FT	0.754**	1	0.476**	0.566**	0.639**
LVLAS	0.330*	0.476**	1	0.506**	0.548**
GCS-fSENC	0.426**	0.566**	0.506**	1	0.686**
GLS-fSENC	0.468**	0.639**	0.548**	0.686**	1

**p < 0.005; *p < 0.05

**Table 6:** Intraclass correlation coefficient for all deformation parameters

	GCS-FT	GLS-FT	LVLAS	GCS-fSENC	GLS-fSENC
GCS-FT	1	0.857**	0.496*	0.576**	0.633**
GLS-FT	0.857**	1	0.644**	0.711**	0.779**
LVLAS	0.496*	0.644**	1	0.653**	0.705**
GCS-fSENC	0.576**	0.711**	0.653**	1	0.806**
GLS-fSENC	0.633**	0.779**	0.705**	0.806**	1

**p < 0.005; *p < 0.05

**Table 7:** Coefficient of variation (CoV) for all deformation parameters (%)

	CoV (%)
GCS-FT versus GCS-fSENC	21.53
GLSL-FT versus GLS-fSENC	18.18
GLS-FT versus LVLAS	26.62
GLS-fSENC versus LVLAS	22.33

Bland–Altman plots revealed similar levels of variability for GLS (fSENC, FT) and LVLAS (CoV 22.33%; CoV 26.62%). Variability was lowest between GLS as derived by fSENC compared to FT (CoV 18.18%).

### Correlation to LVEF, LVESV and LVEDV

We performed a separate analysis regarding the diagnostic accuracy of LVEF, LVESV and LVEDV within our patient cohort. All three values did not allow for significant differentiation between the three patient groups (0: non-cardiac/1: ACS/2: cardiac-non-ACS). Only the LVEDV could significantly distinguish between group 0 and group 2 (*p* < 0.05). In a ROC curve analysis, the functional parameters showed a weak performance with the LVEDV demonstrating the highest AUC amongst them for identification of myocardial dysfunction (LVEF AUC: 0.485; LVESV AUC: 0.583; LVEDV AUC: 0.686). In a further analysis we correlated the different strain values to LVEF and LVESV/LVEDV. All strain parameters (GLS-FT, GCS-FT, GLS-fSENC, GCS-fSENC, LVLAS) correlated significantly (*p* < 0.05) to the LVEDV whereas for LVEF and LVESV it was only GLS-FT and GCS-FT which correlated significantly (*p* < 0.05).

### Inter-observer-/intra-observer variability feature tracking

**Fig. 5:**
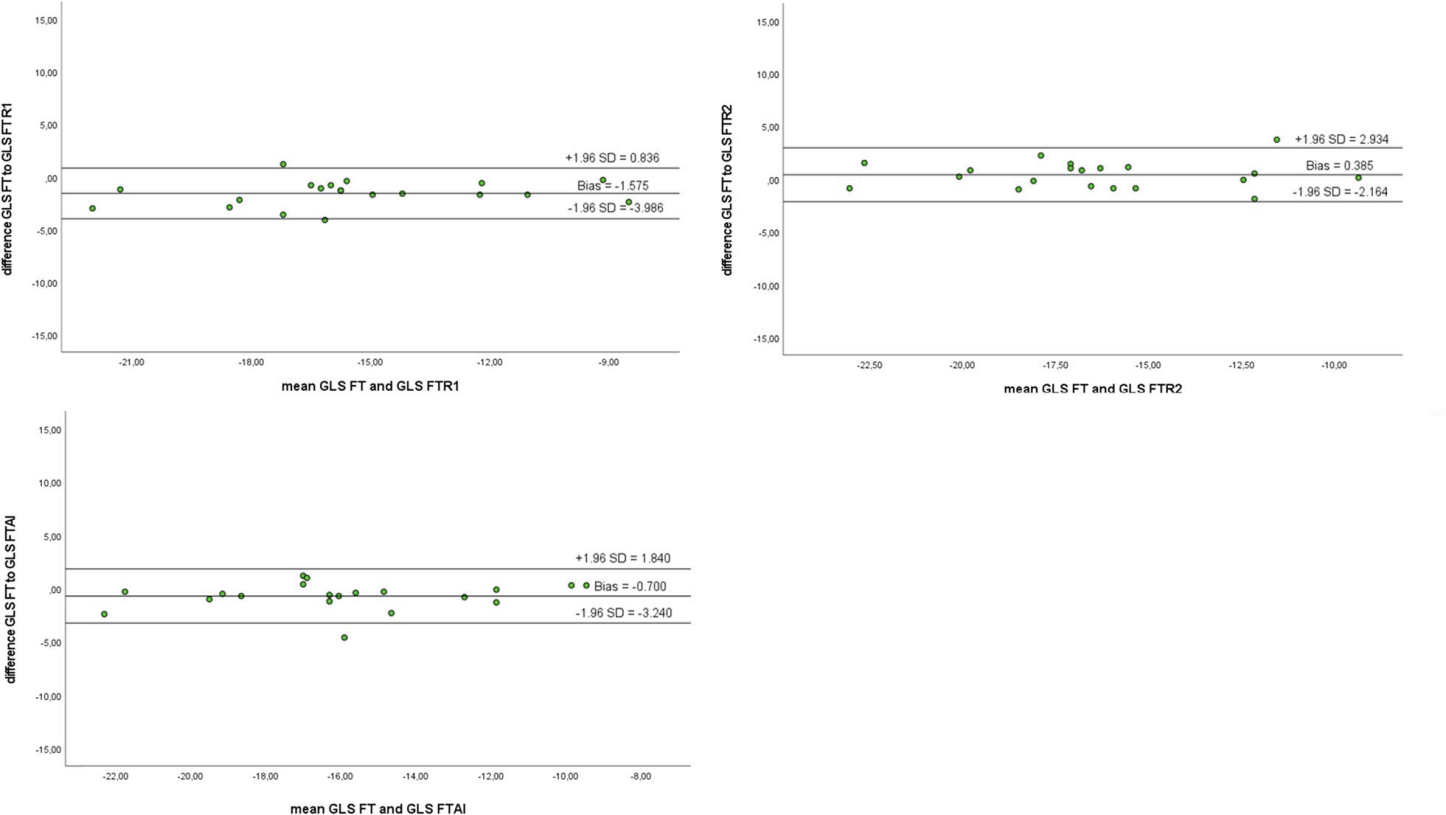
Bland–Altman plots for intra- (reader 1 R1) and inter-observer (reader 2 R2) reliability for GLS values derived by FT. Additional analysis of variability between original GLS values and GLS calculated using artificial intelligence (AI) tools

**Fig. 6:**
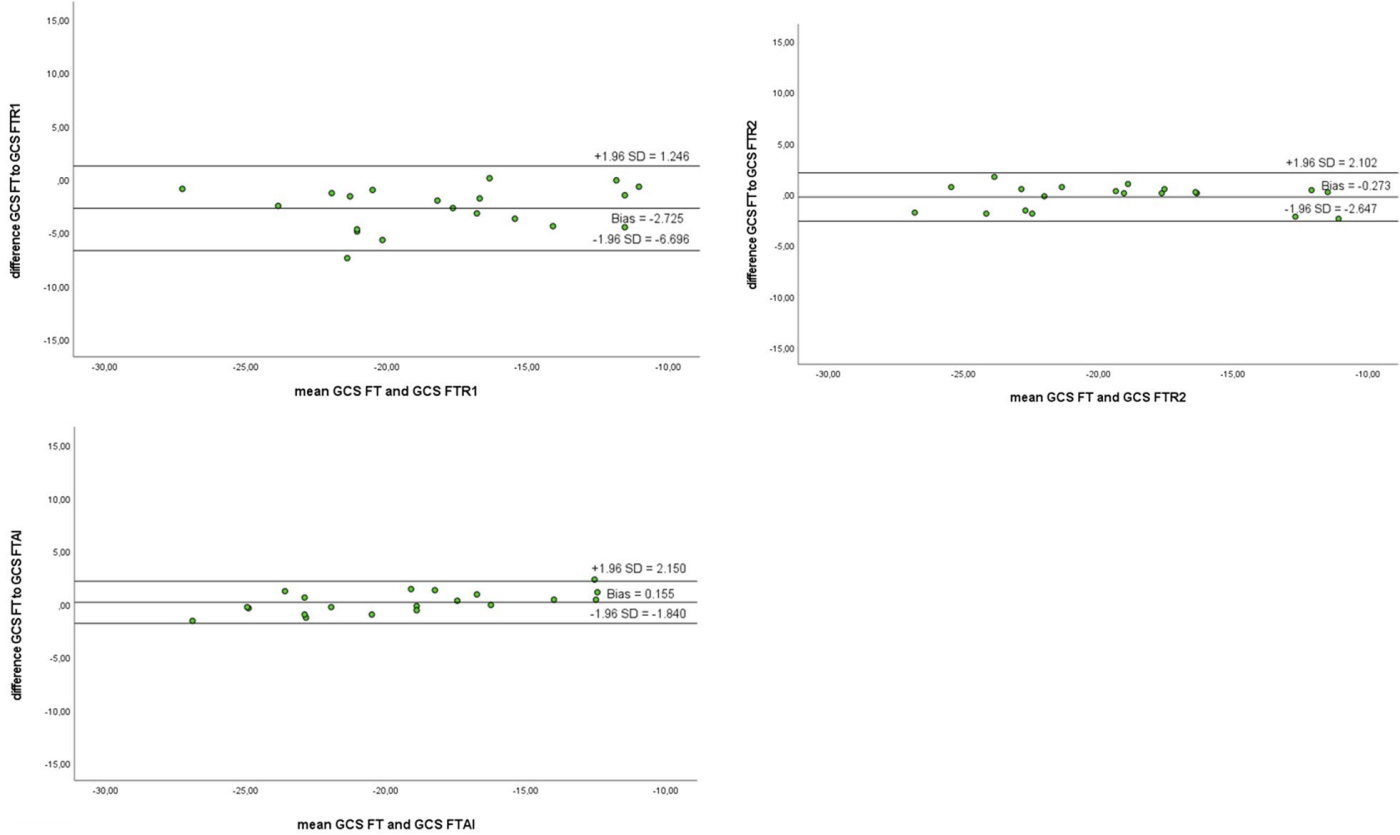
Bland–Altman plots for intra- (reader 1 R1) and inter-observer (reader 2 R2) reliability for GCS values derived by FT. Additional analysis of variability between original GLS values and GLS calculated using artificial intelligence (AI) tools

Intra- and inter-observer reliability was excellent for FT (Pearson and ICC > 0.85). Strain values derived by automated contours using AI tools were similarly reproducible (Pearson and ICC > 0.85). All data were statistically highly significant (*p* < 0.005). Correlation, limits of agreement (LoA), biases and coefficient of variation (CoV) are depicted in Figs. 5, 6 and Tables 8, 9, 10. Variation was lowest between GCS-FT compared to GCS-FT-AI (CoV 5.32%).

**Table 8:** Pearson’s correlation coefficient/Intraclass correlation coefficient for GCS as derived by reader 1 (intra-abserver reliability) and reader 2 (inter-observer reliability) as well as AI (artificial intelligence)

	GCS	GCS R1	GCS R2	GCS AI
GCS	1	0.905**/0.949**	0.968**/0.984**	0.983**/0.987**
GCS R1	0.905**/0.949**	1	0.903**/0.947**	0.874**/0.932**
GCS R2	0.968**/0.984**	0.903**/0.947**	1	0.976**/0.982**
GCS AI	0.983**/0.987**	0.874**/0.932**	0.976**/0.982**	1

**p < 0.005; *p < 0.05

**Table 9:** Pearson’s correlation coefficient/Intraclass correlation coefficient for GLS as derived by reader 1 (intra-abserver reliability) and reader 2 (inter-observer reliability) as well as AI (artificial intelligence)

	GLS	GLS R1	GLS R2	GLS AI
GLS	1	0.942**/0.969**	0.937**/0.967**	0.936**/0.966**
GLS R1	0.942**/0.969**	1	0.890**/0.941**	0.923**/0.960**
GLS R2	0.937**/0.967**	0.890**/0.941**	1	0.936**/0.966**
GLS AI	0.936**/0.966**	0.923**/0.960**	0.936**/0.966**	1

**p < 0.005; *p < 0.05

**Table 10:** Coefficient of variation (CoV) for intra- (reader 1 R1) and inter-observer (reader 2 R2) reliability of GLS and GCS values derived by FT. Additional CoV between original GLS and GCS values and those derived by artificial intelligence (AI) tools

	CoV (%)
GLS-FT versus GLS-FT-R1	8.16
GLS-FT versus GLS-FT-R2	8.35
GLS-FT versus GLS-FT-AI	8.47
GCS-FT versus GCS-FT-R1	10.99
GCS-FT versus GCS-FT-R2	6.36
GCS-FT versus GCS-FT-AI	5.32

### Inter-observer-/intra-observer variability fSENC

**Fig. 7:**
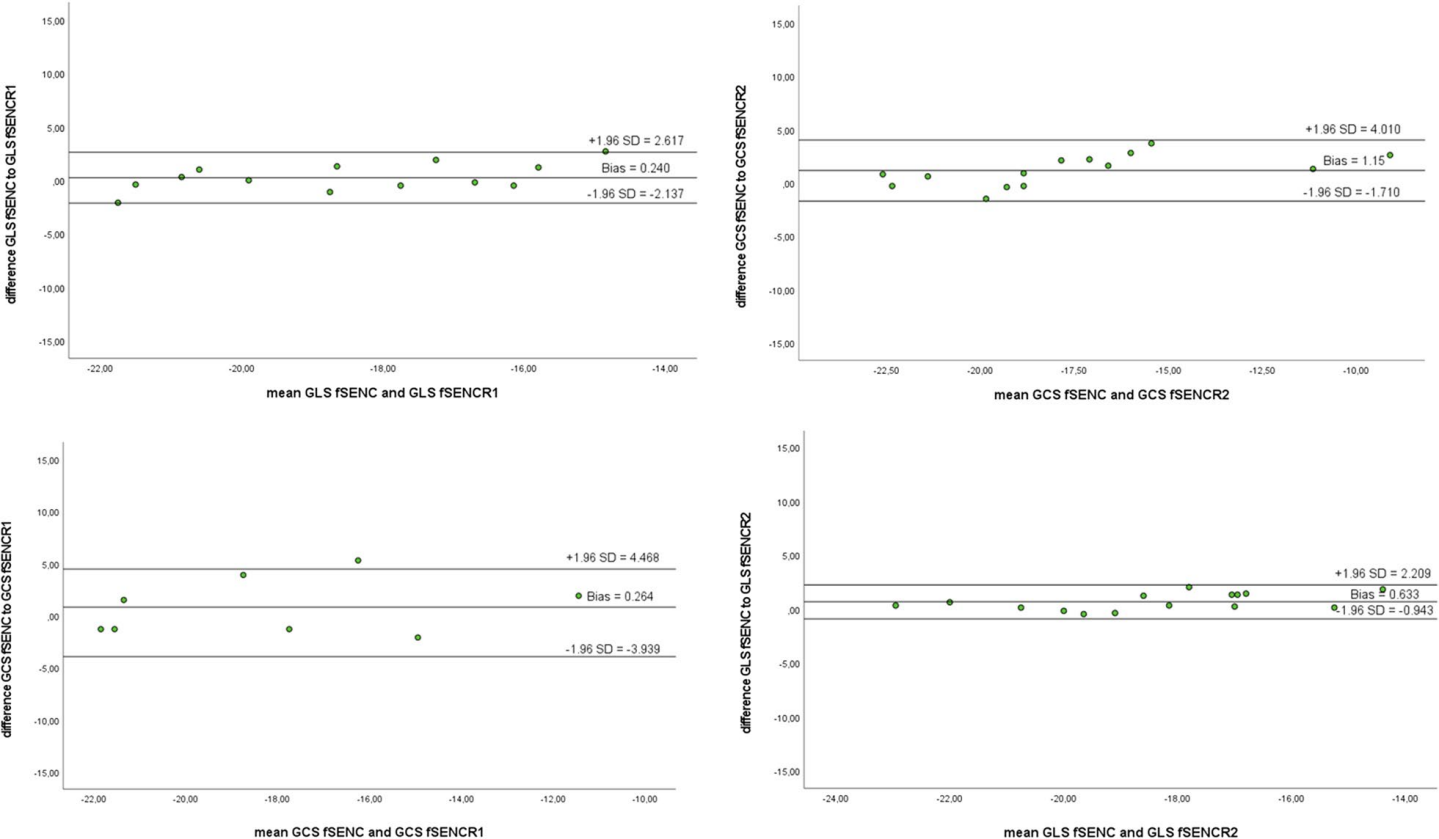
Bland–Altman plots for intra- (reader 1 R1) and inter-observer (reader 2 R2) reliability for GCS and GLS values derived by fSENC

Intra- and inter-observer reliability for fSENC was excellent and comparable to that of FT (Pearson and ICC > 0.8). All data were statistically highly significant (*p* < 0.005). Correlation, limits of agreement (LoA), biases and coefficient of variation (CoV) are depicted in Fig. 7 and Tables 11, 12, 13. Variation was lowest for GLS-values (vs. R1 6.80%; vs. R2 4.36%).

**Table 11:** Pearson’s correlation coefficient/Intraclass correlation coefficient for GCS as derived by reader 1 (intra-abserver reliability) and reader 2 (inter-observer reliability)

	GCS	GCS R1	GCS R2
GCS	1	0.876**/0.934**	0.954**/0.965**
GCS R1	0.876**/0.934**	1	0.859**/0.914**
GCS R2	0.954**/0.965**	0.859**/0.914**	1

**p < 0.005; *p < 0.05

**Table 12:** Pearson’s correlation coefficient/Intraclass correlation coefficient for GLS as derived by reader 1 (intra-abserver reliability) and reader 2 (inter-observer reliability)

	GLS	GLS R1	GLS R2
GLS	1	0.889**/0.927**	0.955**/0.971**
GLS R1	0.889**/0.927**	1	0.818**/0.898**
GLS R2	0.955**/0.971**	0.818**/0.898**	1

**p < 0.005; *p < 0.05

**Table 13:** Coefficient of variation (CoV) for intra- (reader 1 R1) and inter-observer (reader 2 R2) reliability of GLS and GCS values derived by fSENC

	CoV (%)
GLS-fSENC versus GLS-fSENC-R1	6.80
GLS-fSENC versus GLS-fSENC-R2	4.36
GCS-fSENC versus GCS-fSENC-R1	12.43
GCS-fSENC versus GCS-fSENC-R2	8.29

### Inter-observer-/intra-observer variability LVLAS

**Fig. 8:**
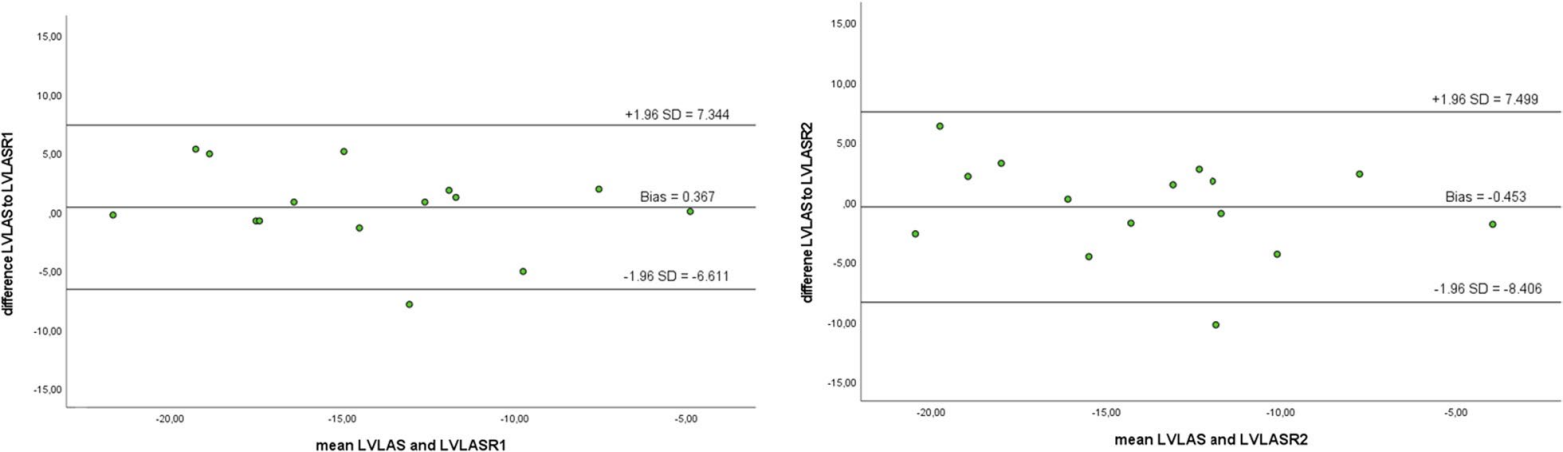
Bland–Altman plots for intra- (reader 1 R1) and inter-observer (reader 2 R2) reliability for LVLAS

While intra-observer reliability was slightly higher (Pearson > 0.7, ICC > 0.8) than inter-observer reliability (Pearson > 0.65, ICC > 0.8), the LVLAS showed lower levels of correlation than FT or fSENC-derived strain values. The correlation data was highly significant (*p* < 0.005). Correlation, limits of agreement (LoA), biases and coefficient of variation (CoV) are depicted in Fig. 8 and Tables 14, 15. It is evident that variation was markedly higher for LVLAS as compared to FT or fSENC with CoV > 25%.

**Table 14:** Pearson’s correlation coefficient/Intraclass correlation coefficient for LVLAS as derived by reader 1 (intra-observer reliability) and reader 2 (inter-observer reliability)

	LVLAS	LVLAS R1	LVLAS R2
LVLAS	1	0.750**/0.850**	0.686**/0.804**
LVLAS R1	0.750**/0.850**	1	0.938**/0.968**
LVLAS R2	0.686**/0.804**	0.938**/0.968**	1

**p < 0.005; *p < 0.05

**Table 15:** Coefficient of variation (CoV) for intra- (reader 1 R1) and inter-observer (reader 2 R2) reliability of LVLAS

	CoV (%)
LVLAS versus LVLAS-R1	25.19
LVLAS versus LVLAS-R2	29.57

## Discussion

CMR strain imaging has gained more widespread attention in recent years and been shown to be highly reproducible [4, 21–24]. Reference LV-strain values for FT and fSENC as well as LVLAS have been previously reported [16, 25]. In direct comparison to echocardiographic speckle tracking CMR-derived strain has been shown to correlate significantly to echocardiographic data while being less user-dependent and less subject to suboptimal sonographic conditions [26].

Nevertheless, widespread clinical applicability is hampered by the lack of rapid access to MRI scanners in most institutions [27], a multitude of techniques for strain evaluation [28], and poor inter-vendor agreement [29–31]. Additionally, strain values still vary between methods, modalities, and software versions [32] and still lack proper validation [33].

Therefore, more studies regarding feasibility and reproducibility of different CMR-based strain techniques are needed to allow for standardization and subsequently more widespread clinical utilization of myocardial strain.

In our study we directly compared three different methods for assessment of myocardial deformation (FT/fSENC/LVLAS) within a study population of patients with chest pain.

The main findings of our study are the following:

1. LVLAS was comparable to fSENC-derived strain for differentiation between healthy myocardium (non-cardiac chest pain) and myocardial dysfunction (ACS and underlying cardiac pathology) while FT-derived strain showed the weakest performance.
2. GLS-fSENC was the only parameter which allowed for significant patient triage according to final diagnosis (non-cardiac, ACS, cardiac-non-ACS).
3. There was significant variability between the three techniques with moderate correlation of GCS and GLS (FT/fSENC) to LVLAS.
4. Functional parameters (LVEF, LVESV, LVEDV) were less susceptible than strain for identification of cardiac pathologies.
5. Intra- and inter-observer agreement and variability of global strain values were excellent for FT and fSENC.
6. LVLAS showed lower levels of intra- and inter-observer agreement and higher variability than FT or fSENC.

LVLAS is a relatively new method for assessing global left ventricular function – allowing significant discrimination of patients with cardiomyopathies from healthy subjects [13]. Although it has already been shown to predict cardiac events in patients with non-ischemic dilated cardiomyopathy [34], no studies to date have assessed LVLAS performance within an ischemic patient population. Furthermore, no direct comparison of LVLAS to other parameters of myocardial deformation such as global strain has been performed. Reference values have been previously established and set at −17.1 ± 2.3%. Of note, mean values were significantly higher for women and younger people [13]. In our study cohort the LVLAS values were higher (−13.42 ± 3.87%). This may be explained by our relatively young patient population (mean age 57.1 ± 17.7 years).

Interestingly, within our study population LVLAS allowed for similar differentiation between cardiac pathology and non-cardiac disease as fSENC-derived global strain values and demonstrated a better performance than FT. ROC curves did not differ significantly from each other due to the small sample size of patients deemed to be suffering from cardiac pathology and need to be evaluated in a bigger patient population. This data is promising and highlights the usefulness of LVLAS as a rapid alternative approach for the assessment of ventricular function.

FT-derived regional strain has been shown to allow differentiation between areas of myocardial scar and healthy myocardium [35]. We were able to confirm this in our study. However, fSENC-derived global strain as well as the LVLAS proved to be superior to FT-derived global strain for differentiation between healthy and impaired myocardium within our study population. Overall, GCS-fSENC provided a higher accuracy for identification of cardiac dysfunction (AUC: 0.899). However, GLS-fSENC was the only parameter which further allowed for patient triage according to final clinical diagnosis. This is in line with previous studies that have shown GLS to be a feasible alternative to LVEF for the evaluation of myocardial function and risk stratification [36, 37]. Furthermore, GLS has been investigated within a study population of patients following trans-aortic valve replacement and patients undergoing cardiotoxic chemotherapy with good prognostic performance [38, 39].

Correlation between the three modalities was moderate with high variability. GLS-FT and GLS-fSENC showed the best correlation (Pearson > 0.5, ICC > 0.75) with the least variation (CoV = 18.18%). This confirms previous data showing that differences in image acquisition and post-processing analysis may ultimately lead to substantial bias [40, 41]. The fixed position of slices renders FT susceptible to through-plane motion artifacts. fSENC on the other hand, depends on the correct orthogonal positioning of the tag lines with the need for meticulous image planning. More studies are needed to establish validated standardized reference values before fSENC- and FT-global strain data can be rendered comparable.

In general, functional parameters such as LVEF, LVESV and LVEDV per se did not allow for significant identification of the underlying cardiac pathologies within our study cohort. This is in line with previous studies which have indicated that strain is a more sensitive marker of early left ventricular dysfunction than left-ventricular ejection fraction (LVEF) [42, 43]. Interestingly, only the LVEDV allowed for significant differentiation between healthy patients and those suffering from an underlying cardiac disease. This makes sense, as HCM or hypertensive heart disease typically initially present with diastolic dysfunction and only lead to reduced EF in later stages of the disease [44, 45]. All strain parameters correlated significantly only to the LVEDV which underlines the fact that strain as well as the LVEDV were the more susceptible markers for earlier identification of cardiac pathologies within our study cohort.

Intra- and inter-observer analysis was excellent for both FT and fSENC with low levels of variation. However, correlation was lower for LVLAS intra-/inter-observer reliability with higher variation. This stands in contrast to the previous data by Riffel et al. which showed low levels of variability (intra-OV: 5.6 ± 4.2%, inter-OV: 6.3 ± 4.2%) within a larger patient cohort consisting of 40 healthy volunteers and 125 cardiomyopathy patients [13]. This discrepancy may be explained by our smaller sample size (15 patients) leading to a potential larger impact of outliers we were able to observe. Nevertheless, it needs to be noted that the correct calculation of LVLAS is susceptible to several factors such as the correct identification of end systolic and end diastolic phases of the cardiac cycle as well as the accurate definition of the mitral valve ring. We believe with correct training and experience these difficulties can be overcome and LVLAS may be used as a supporting or in certain cases, alternative parameter for assessment of LV function. More studies evaluating accuracy and reproducibility of LVLAS within larger patient samples are required.

### Limitations

The main limitation of our study is the relatively small sample size. Additionally, findings were not compared to standard CMR protocols or echocardiographic derived strain data. No extended follow-up was performed—prospective studies are required to evaluate LVLAS as a prognostic parameter.

## Conclusions

While reproducibility was excellent for both FT and fSENC. It was only fSENC and the LVLAS which allowed for significant identification of myocardial dysfunction, even with preserved LVEF, and therefore might be used as additional parameters for the assessment of left-ventricular function.

## Data Availability

The datasets generated and/or analysed during the current study are not publicly available as the contained information could compromise the privacy of research participants, but are available from the corresponding author on reasonable request.

## Abbreviations

ACS: Acute coronary syndrome
AI: Artificial intelligence
AUC: Area under the curve
BMI: Body mass index
BP: Blood pressure
CMR: Cardiac magnetic resonance
COV: Coefficient of variation
CT: Computer tomography
ECG: Electrocardiogram
EDV: Enddiastolic volume
ESV: Endsystolic volume
FOV: Field of view
fSENC: Fast strain encoded
FT: Feature tracking
GCS: Global circumferential strain
GLS: Global longitudinal strain
hscTnT: High-sensitivity cardiac troponin T
ICC: Intraclass correlation coefficient
IOV: Interobserver variability
LAX: Long axes
LoA: Limits of agreement
LVEF: Left ventricular ejection fraction
LVLAS: Left ventricular long axis strain
NYHA: New York Heart Association
Py: Pack years
ROC: Receiver operating curve
SAX: Short axes
SSFP: Steady-state free precession
TE: Time to echo
TR: Time to repeat
